# Nursing students and role modeled behavior while caring for LGBTQ + people: a cross-sectional, descriptive study

**DOI:** 10.1186/s12912-024-02618-0

**Published:** 2024-12-21

**Authors:** Emily E. Roy, Kristen D. Clark

**Affiliations:** 1 Department of Nursing, University of New Hampshire, Hewitt Hall, 4 Library Way, Durham, NH USA; 2 Department of Medical Sciences, Uppsala University, Akademiska Sjukhuset, Ingång 10, Plan 3, Uppsala, 751 85 Sweden

**Keywords:** LGBTQ +, Gender identity, Sexual and gender minority, Nursing students, Education

## Abstract

**Background:**

While efforts to improve the educational preparedness of nurses to care for lesbian, gay, bisexual, transgender, and queer (LGBTQ +) people have increased, the influence of role-modeled behaviors by healthcare professionals working with nursing students and recent graduates is not well understood. The purpose of this study is to describe the role-modeled behaviors of healthcare professionals observed by nursing students and recent graduates caring for LGBTQ + patients in clinical settings.

**Methods:**

A cross-sectional, online survey was conducted. Recruitment of nursing students who had completed one or more clinical rotations or were recent graduates (≤ 2 years) was performed through university emails and social media. Items included measurement of stigmatizing attitudes, observed stigmatizing behaviors, and ability to provide inclusive/affirming care for LGBTQ + patients. Open-text items prompted participants to describe observed behaviors. Data were analyzed using descriptive statistics and Wilcoxon signed rank sum tests to evaluate differences between LGB (lesbian, gay, bisexual) and T + (transgender and gender diverse) subscales. Open-text responses were analyzed using thematic analysis to identify relevant themes.

**Results:**

Participants (*N* = 73) had a low level of stigmatizing attitudes toward LGBTQ + people (M = 1.8, SD = 0.4), although higher stigmatizing attitudes toward T + people were reported (M = 3.0, SD = 0.2; *Z* = -7.254, *p* < .001). Half of the participants reported that they observed LGBTQ + stigmatizing behaviors role-modeled by two + healthcare professional roles; approximately one-third of participants personally engaged in one + LGBTQ + stigmatizing behaviors, most commonly toward T + people. Themes from participants’ examples of observed stigmatizing behaviors included: *cis-heteronormative bias*, *non-affirmation of chosen name/pronouns*, *outing patients*, and *rejected competency*.

**Conclusions:**

The majority of participants described observing stigmatizing behaviors toward LGBTQ + people in clinical settings. Poorer attitudes and a higher frequency of stigmatizing behaviors observed towards T + people point to deficits in healthcare provided to T + people in particular. Efforts to address LGBTQ + stigma in healthcare should be expanded to include clinical settings to address role-modeled behaviors and socialization of nurses.

## Background

The proportion of people globally who identify as lesbian, gay, bisexual, transgender, queer, and other diverse sexual and gender identities (LGBTQ +) has grown steadily in recent years [1–3]. As this population grows, so does the need for accessible healthcare that is comprehensive and culturally inclusive. Historically, LGBTQ + people have experienced stigmatization and discrimination due to their sexual orientation or gender identity in all areas of society, including healthcare [4].

When seeking care, LGBTQ + people encounter healthcare systems that are not built to accommodate diverse sexual and gender identities, as well as healthcare professionals who have had limited educational preparation to effectively deliver inclusive and affirming LGBTQ + care [5]. People who are lesbian, gay, bisexual, or other diverse sexualities (LGB) encounter heteronormativity, (*i.e*., the default assumption that all people are heterosexual). Heteronormativity manifests as healthcare providers’ assumptions about the gender of patients’ partners as well as stigmatizing behaviors (*e.g*., excluding LGBTQ + partners from visiting), resulting in a lower quality of care [5, 6]. Transgender and people with diverse gender identities (T +) report similar heteronormative and stigmatizing experiences but also report instances of misgendering (*i.e*., referring to a person by incorrect pronouns or sex, such as using he/him pronouns for a transgender woman who uses she/her), deadnaming (*i.e.*, referring to a patient by the name they were given at birth instead of their chosen name), and denial of healthcare services [7, 8]. LGBTQ + people broadly describe having to educate healthcare professionals on their healthcare needs as related to their sexual orientation and/or gender identity [5–8].

Stigmatization in healthcare is one of many forms of stress experienced by LGBTQ + people that can lead to the poor health outcomes observed in this population. This process has been described as minority stress, where proximal stressors (*e.g*., internalization processes and anticipation of mistreatment) occur due to the marginalization of diverse sexual and gender identities and distal stressors (*e.g*., experiences of stigma; [9–11]). Exposure to distal stressors is associated with poor health outcomes, including an increased risk of substance use disorders [12, 13], disordered eating [14], depression [15, 16], poor physiological health [17–19], and other adverse conditions [20]. Proximal stressors, such as delaying or avoiding healthcare services due to previous experiences of stigmatizing behaviors or anticipation of such behavior, are also associated with poor health outcomes [21–23]. These multifactorial challenges have resulted in the need for inclusive and affirming LGBTQ + practices and systems in healthcare to advance health equity for LGBTQ + people.

One pathway to improve the healthcare experiences of LGBTQ + people is through the education of healthcare professionals. Education on inclusive and affirming care for LGBTQ + people has been integrated into numerous areas of healthcare education, such as simulations and lectures on LGBTQ + health outcomes, to promote the best health outcomes for LGBTQ + patients [24–26]. Despite those advances in education, LGBTQ + people continue to report mistreatment in healthcare settings. For example, a survey conducted in 2022 of over 90,000 T + people in the United States (US) found that almost half of participants experienced mistreatment in healthcare during the past twelve months [27], an increase from the previous survey conducted in 2015 [28]. A 2022 survey by the Center for American Progress found that one out of every five LGBTQ + people experienced mistreatment in healthcare during the past year, and one out of every three reported mistreatment in mental healthcare settings [20]. These findings indicate that further efforts to improve LGBTQ + healthcare experiences are needed. One pathway to improvement is to examine the education of healthcare professionals outside of the classroom and evaluate the socialization process in clinical training environments.

Clinical environments are a space where pre-licensure healthcare professionals apply skills and gain knowledge of real-world contexts. These can also be the environments where LGBTQ + people encounter mistreatment. In these settings, students practice their required skills, but they are also educated through role-modeling by professionals to acclimate within the social norms both within their respective careers and within the healthcare environment. Bandura’s Social Learning Theory describes how an individual acquires knowledge from their background, their education, and their observations of the people who surround them [29]. A key concept involves mimicking others’ behaviors and the reinforcement of those extrinsic behaviors. While curricula aimed at providing education on the use of LGBTQ + inclusive and affirming practices pose an opportunity to improve care delivery, the behavior modeled to students during clinical experiences should also be considered. Students’ experiences working with healthcare professionals can either support the implementation of knowledge gained from curricula or be in conflict, potentially encouraging undesirable behavior when caring for LGBTQ + people. In addition to the role-modeled behavior itself, there is an imbalance of power, where students defer to practicing healthcare professionals as knowledgeable authority figures. This can reinforce the learning of incorrect behaviors and may also inhibit students’ and recent graduates’ willingness to speak out when witnessing patient mistreatment. One study of patient weight bias among medical students found that students who observed their faculty or mentors making weight-stigmatizing statements were more likely to hold negative beliefs about people in larger bodies [30, 31]. This association has also been observed in care delivery, as medical students who observed weight-stigmatizing behaviors on the part of faculty were found to provide a lower quality of care during a standardized patient simulation [32]. Compared to medical students, the relationship between role-modeling and patient care among nursing students and recent graduate nurses, specifically, has been understudied. Previous studies have found that nursing students and recent graduates’ behaviors are influenced by preceptors and other nurses; however, social learning related to LGBTQ + patients specifically was not examined [33–35]. While curricular interventions are needed to improve nursing student knowledge and preparedness to care for LGBTQ + people [25, 26], the influence of role modeling in clinical settings has not been adequately studied. The purpose of this study was to describe the role-modeled behaviors observed by nursing students and recent graduate nurses related to the care of LGBTQ + patients in clinical settings.

## Methods

### Recruitment

Participants were recruited via convenience sampling school email distribution through a university in the northeast US and nationally through social media (*i.e.*, Instagram, Facebook). Approximately 80 students are enrolled each year in the nursing program where the study was conducted. In 2023, approximately 255,000 students were enrolled in undergraduate nursing programs in the US [36]. Students were provided a link to the Qualtrics survey from February 15 to March 15, 2023. Upon survey completion, participants were directed to a separate Qualtrics link where they could enter a raffle for a $50 Visa gift card.

### Inclusion criteria

To be included in the study, participants had to be 18 years or older, be enrolled in a prelicensure nursing program (*i.e*., associate’s, bachelor’s, or graduate entry-to-practice) in the northeastern US, and have completed one clinical rotation. Students who had recently graduated and had worked as registered nurses for two years or less were also eligible*.* Both current students and recent graduates were included as both groups are novices in nursing practice and vulnerable to the differential in power and authority held by more experienced healthcare professionals with whom they closely work.

### Study methodology

A cross-sectional questionnaire was created to measure participant demographics, attitudes, observations of role-modeled behaviors, and experiences using inclusive and affirming healthcare practices related to LGBTQ + patients and patients living in larger bodies [35]. The survey took approximately 30 min to complete. The present study analyzed only the survey responses related to LGBTQ + people.

### Clinical trial number

Not applicable.

### Demographic measures

Demographics measured included age, gender identity, sexual orientation, race, ethnicity, and characteristics of nursing education. Age was measured by a categorical variable in ranges of 18–25, 26–32, 33–39, and 40 + . Gender identity was measured by a select-all-that-apply variable with the following answer choices: man, non-binary or genderqueer, woman, or gender not listed. Sexual orientation was measured by a select-all-that-apply variable with the following answer choices: asexual, heterosexual/straight, gay/lesbian, pansexual/bisexual, queer, or a sexual orientation not listed. Participants were asked, “Do you have close friends or family members who are part of the LGBTQ + community?” and could respond yes or no.

### Stigmatizing attitudes toward LGBTQ + people

Three separate scales were adapted to a brief 14-item measure to assess stigmatizing attitudes toward LGBTQ + people: Utilizing the Attitudes Towards Lesbians and Gay Men [37], the Attitudes Towards Bisexual Men and Women [38], and the Attitudes Towards Transgender Men and Women [39]. All scales have shown high internal consistency in previous studies (α ≥ 0.91) [37–39]. Participants could indicate on a Likert-type scale the degree to which they agreed or disagreed with the statements about LGBTQ + people with eight reverse-coded items. Items were summed and divided by the number of items for total LGBTQ + attitudes (14 items) and also separately for LGB (10 items) and T + (4 items) attitudes, providing three variables with a range from 1–5, where a higher number indicates greater stigmatizing attitudes.

### Observations of LGBTQ + stigma

Observations of LGBTQ + stigma were measured by an item that asked “Which of the following have you observed [healthcare professional role] engaging in at clinical/healthcare setting when caring for an LGBTQ + patient?” Operational definitions and examples were provided for each type of stigma (Table 1). Participants could then indicate any LGBTQ + stigmatizing behaviors they have observed with seven types of LGBTQ + stigma. Participants were then prompted with the same question four times for the following healthcare professional roles: “clinical instructor”, “nurse or nursing assistant”, “provider or other healthcare professionals”, and “you” (referring to the participant themselves).

**Table 1:** Key terms related to the present study as presented to participants in the survey

Term	Definition Provided to Participants
Transgender	Individuals whose gender identity does not align with the sex they were assigned to at birth. For the purpose of this study, it is used as an umbrella term to include non-binary people and genderqueer people
Gender-affirming care	Practices that convey acceptance and respect of a person’s gender identity
Deadnaming	Using the name that was given to a transgender person at birth when they have chosen a new name that represents their gender. For example, if a person was referred to as Susan but has asked to be called Forrest. This could be deliberate or accidental
Misgendering	Using pronouns to refer to a person contrary to how that person wishes to be described. For example, referring to a transgender woman as “he” and “a man”. This could be deliberate or accidental
Homophobic	Actions, statements, or behaviors that communicate a dislike or disdain for people who are not heterosexual (straight), or a preference for people who are heterosexual (straight)
Transphobic	Actions, statements, or behaviors that communicate a dislike or disdain for people who are transgender, or a preference for people who are not transgender

Participants were also provided an open-text item to provide additional details about any LGBTQ + stigma they observed in clinical settings. Participants were prompted, “Please describe any events or experiences that will help us understand or more clearly illustrate your responses above. Please be cautious not to report any specifics that could disclose patient identities or violate HIPAA (*e.g.*, names of hospitals, patient names).”

### Analysis

Gender identity and sexual orientation were combined to create a single variable indicating whether a participant identified as LGBTQ + to reduce the potential for identification of individual participants. To account for variations in nursing program structure (e.g., associate’s degree, bachelor’s degree, and graduate entry-to-practice programs), participants were categorized based on their stage in the program. Those in the initial half of their program were categorized as early-stage learners, while those in the latter half were categorized as late-stage learners. This classification reflects their relative progression toward program completion and clinical readiness. Internal consistency among survey items was examined using Cronbach’s alpha. Descriptive statistics were used to describe participant characteristics and study outcomes across the total group and also by subgroup (i.e., current students, including early- and late-stage learners, and recent graduates). Inferential statistics (*i.e.*, Wilcoxon signed rank sum test) were used to examine differences in participant responses as they related to specific subpopulations (*i.e*., LGB and T +). Statistical analyses were conducted using STATA version 15.1 [40]. Open-text responses were analyzed using thematic analysis, employing an inductive approach and line-by-line coding by two independent coders [41]. Representative quotes were identified to best illustrate the themes.

## Results

### Demographics

Seventy-three participants completed the survey. The majority were cisgender, heterosexual people (*n* = 65, 89.0%) and white non-Hispanic (*n* = 68, 91.8%). Eight (11%) of participants identified as LGBTQ + . Approximately 90% were current students (*n* = 65), and the majority were currently enrolled in (or recently graduated from) a 4-year prelicensure nursing program (*n* = 67, 91.8%). The majority of currently enrolled students were late-stage learners (*n* = 36, 49.3%). Only 10% of participants (*n* = 8) were recent graduates. Almost 90% of participants (*n* = 64) indicated that they had close friends or family who identified as LGBTQ + .

### Stigmatizing attitudes toward LGBTQ + people

Items measuring attitudes toward LGBTQ + people showed strong internal consistency: total stigmatizing LGBTQ + attitudes (α = 0.93), LGB subscale (α = 0.90), and T + subscale (α = 0.91). Among participants, stigmatizing attitudes toward LGBTQ + people overall were low (M = 1.8, SD = 0.4). However, stigmatizing attitudes toward T + people (M = 3.0, SD = 0.2) were notably higher when compared to LGB people (M = 1.3, SD = 0.6; *Z* = −7.254, *p* < 0.001). There was little variation in these scores when evaluated within sample subgroups. Table 2 provides full demographic characteristics and stigmatizing LGBTQ + attitudes in the total sample and subgroups can be found in Table 3.

**Table 2:** Participant demographic results (N = 73)

Demographic Characteristic	N (%)
Age	
18–25 years	67 (91.8)
26 + years	6 (8.2)
Sexual Orientation and Gender Identity^a^	
Cisgender & heterosexual/straight	65 (89.0)
LGTBQ +	8 (11.0)
Race and Ethnicity^a^	
White	67 (91.8)
Racial and/or ethnic minority	6 (8.2)
Student Status	
Current student	65 (89.0)
Recent graduate	8 (11.0)
Type of Nursing Program, Current or Recently Graduated From	
2-year Associates	3 (4.1)
4-year Bachelors	67 (91.8)
Other^b^	3 (4.1)
Year in Nursing Program (Current Students Only)	
2^nd^	32 (43.8)
3^rd^	13 (17.8)
4th	19 (26.0)
Other^b^	1 (1.4)
Not reported	8 (11.0)

^a^Participants could select all options that apply, therefore patients could be reflected in more than one category for this item

^b^Accelerated BSN or other graduate entry to practice

**Table 3:** Frequencies of LGBTQ + stigmatizing behaviors in clinical settings by sample subgroup

**Outcome**	**Total Sample**	**Current Students**			**Recent Graduates**
		All Students	Early-stage learner	Late-stage learner	
	*n* = 73	*n* = 65	*n* = 29	*n* = 36	*n* = 8
	M (SD)	M (SD)	M (SD)		M (SD)
LGBTQ + Stigmatizing Attitudes	1.8 (0.4)	1.8 (0.5)	1.8 (0.3)	1.8 (0.6)	1.7 (0.1)
LGB stigmatizing attitudes	1.3 (0.6)	1.3 (0.6)	1.2 (0.4)	1.4 (0.8)	1.1 (0.2)
T + stigmatizing attitudes	3.0 (0.2)	3.0 (0.2)	3.1 (0.2)	3.0 (0.2)	3.1 (0.2)
	*p* < .001	*p* < .001	*p* < .001	*p* < .001	*p* < .01
	*n* (%)	*n* (%)	*n* (%)	*n* (%)	*n* (%)
Types LGBTQ + Stigmatizing Behaviors Observed^a^					
Deadnaming a patient	20 (27.4)	16 (24.6)	2 (6.9)	14 (38.9)	4 (50.0)
Derogatory comments about a patient’s sexual orientation or gender identity	9 (12.3)	9 (13.9)	3 (10.3)	6 (16.7)	0 (0)
Homophobic comments	11 (15.1)	8 (12.3)	2 (6.9)	6 (16.7)	3 (37.5)
Misgendering a patient	28 (38.4)	21 (32.3)	4 (13.8)	17 (47.2)	7 (87.5)
Shared patient’s sexual orientation/gender identity to people who are not part of their care team	14 (19.2)	10 (15.4)	0 (0)	10 (27.8)	4 (50.0)
Transphobic comments	22 (30.1)	19 (29.2)	3 (10.3)	15 (44.4)	3 (37.5)
Unequal treatment	7 (9.6)	6 (9.2)	2 (6.9)	4 (11.1)	1 (12.5)
Engaged in at least one stigmatizing behavior					
Participant	23 (31.5)	18 (27.7)	2 (6.9)	16 (44.4	5 (62.5)
Instructor		13 (20.0)	1 (3.4)	12 (33.3)	2 (25.0)
Nurse		33 (50.8)	8 (27.6)	25 (69.4)	7 (87.5)
Other healthcare professionals	31 (42.5)	25 (38.5)	6 (20.7)	19 (52.8)	6 (75.0)
Engaged in two or more stigmatizing behaviors					
Participant	8 (11.0)	7 (10.8)	0 (0)	7 (19.4)	1 (12.5)
Instructor		7 (10.8)	0 (0)	7 (19.4)	1 (12.5)
Nurse	26 (35.6)	20 (30.8)	4 (13.8)	16 (44.4)	6 (75.9)
Other healthcare professionals	17 (23.3)	14 (21.5)	4 (13.8)	10 (27.8)	3 (37.5)
Number of healthcare professional roles engaging in stigmatizing behavior					
Two or more healthcare professional role	40 (54.8)	33 (50.8)	8 (27.6)	25 (69.4)	7 (87.5)
All healthcare professional roles	5 (10.0)	5 (10.6)	1 (4.0)	4 (18.2)	0 (0)

^a^Totaled across healthcare professional roles, excluding personal behaviors

### Observations of LGBTQ + stigmatization, quantitative findings

Current students who were late-stage learners and recent graduates reported observing the highest proportion of LGBTQ + stigmatizing behaviors. The most commonly observed behaviors were “misgendering a patient” (47.2%, *n* = 17; 87.5%, *n* = 7, respectively), “deadnaming a patient” (38.9%, *n* = 14; 50%, *n* = 4, respectively), and “transphobic comments” (44.4%, *n* = 15; 37.5%, *n* = 3, respectively). Among current students, 20% (*n* = 13) reported observing at least one type of LGBTQ + stigmatizing behavior by a clinical instructor, with these observations most commonly reported by late-stage learners (33.3%, *n* = 12). In contrast, a quarter (*n* = 2) of new graduates reported observing at least one type of behavior. Overall, 20% (*n* = 15) of participants reported at least one type of stigmatizing behavior by a clinical instructor, and almost 11% (*n* = 8) reported two or more. Reports of observing multiple behavior types were limited to late-stage learners ( 11.8%, *n* = 7) and new graduates (12.5%, *n* = 1). The most commonly observed stigmatizing behavior by clinical instructors was “making transphobic comments” (*n* = 14, 19.2%), followed by “deadnaming a patient” (11%, *n* = 8).

Table 4 presents the full results of observed LGBTQ + stigmatizing behaviors by healthcare professional role (*i.e*., participants themselves, clinical instructor, nurse or nursing assistant, and provider or other healthcare team member). Table 3 presents these results in the total sample and by subgroup.

**Table 4:** Frequencies of LGBTQ + stigmatizing behaviors in clinical settings by healthcare professional role

Stigmatizing Behavior	Individual(s) Who Engaged in Stigmatizing Behaviors			
	Participant	Clinical Instructor	Nurse or nursing assistant	Provider or other professional
Deadnaming a patient	8 (11.0)	8 (11.0)	16 (21.9)	16 (21.9)
Derogatory comments about a patient’s sexual orientation or gender identity	0 (0.0)	1 (1.4)	9 (12.3)	5 (6.9)
Homophobic comments	0 (0.0)	2 (2.7)	10 (13.7)	5 (6.9)
Misgendering a patient	18 (24.7)	0 (0.0)	27 (37.0)	22 (30.1)
Shared patient’s sexual orientation or gender identity to people who are not part of their care team	0 (0.0)	3 (4.1)	12 (16.4)	5 (6.9)
Transphobic comments	6 (8.2)	14 (19.2)	20 (27.4)	11 (15.1)
Unequal treatment	0 (0.0)	0 (0.0)	6 (8.2)	3 (4.1)

Similarly, when observing nurses or nursing assistants, more than half (*n* = 33) of current students, particularly later-stage learners (69.4%), reported at least one type of LGBTQ + stigmatizing behavior. Recent graduates reported observing such behaviors even more frequently, with 87.5% (*n* = 7) identifying at least one type of stigmatizing behavior. Across the sample, 54.8% (*n* = 40) reported observing at least one type of behavior, and over a third (*n* = 26) reported observing two or more behavior types. Reports of observing multiple behavior types were most frequent among late-stage learners (44.6%, *n* = 16) and new graduates (75.9%, *n* = 6). The most commonly observed stigmatizing behavior by nurses or nursing assistants was “misgendering a patient” (37%, *n* = 27) closely followed by “making transphobic comments” (27.4%, *n* = 20).

While observing providers or other healthcare professionals, 38.5% (*n* = 25) of current students observed at least one type of stigmatizing behavior. Similar to other healthcare professional roles, this was most commonly observed by late-stage learners (52.8%, n = 9) and recent graduates (75%, *n* = 6). In total, over 40% of participants (*n* = 31) reported observing at least one type of stigmatizing behavior, with almost a quarter (*n* = 17) reporting two or more. Reports of observing multiple behavior types were most frequent among late-stage learners (27.8%, *n* = 10) and new graduates (37.5%, *n* = 3) as well. Participants reported that the most commonly observed types of LGBTQ + stigmatizing behaviors by providers and other healthcare professionals were “misgendering a patient” (30.1%, *n* = 22) and “deadnaming a patient” (21.9%, *n* = 16).

This study also analyzed whether nursing students or recent graduate nurses observed LGBTQ + stigmatizing behavior from multiple healthcare team roles (*i.e*., clinical instructor, nurses or nursing assistants, and providers or other healthcare professionals). Approximately half of current students (*n* = 33) observed stigmatizing behavior by multiple healthcare team roles, with the highest proportion reported by late-stage learners (69.4%, n = 25). Similarly, 87.5% of recent graduates (*n* = 7) observed two or more healthcare team roles engaging in stigmatizing behaviors. Across the sample, 54.8% of participants observed LGBTQ + stigmatizing behaviors from two or more healthcare team roles.

When asked to indicate types of LGBTQ + stigmatizing behaviors they have *personally* engaged in during clinical practice, 27.7% of current students (*n* = 18) indicated at least one type of stigmatizing behavior, the highest proportion among later-stage learners (44.4%, *n* = 16). Almost two-thirds of recent graduates (*n* = 5) also reported that they had engaged in at least one type of stigmatizing behavior. Among the total sample, 31.5% (*n* = 23) of participants described at least one type of stigmatizing behavior, and 11% (*n* = 8) indicated two or more types of LGBTQ + stigmatizing behaviors. The most common type of LGBTQ + stigmatization reported was “misgendering a patient” (24.7%, *n* = 18) which was followed by “deadnaming a patient” (11%, *n* = 8).

### Observations of LGBTQ + stigmatization, qualitative findings

Participants were prompted, “Please describe any events or experiences that will help us understand or more clearly illustrate your responses above.” The themes identified included: *cis-heteronormative bias*, *non-affirmation of chosen names and pronouns*, *outing patients*, and *rejected competency*.

#### Cis-heteronormative bias

This theme is defined as instances where cisgender and heterosexual identities are communicated to be the default and “normal,” and LGBTQ + identities and expressions are patronized or mocked. One participant described,

“I had one patient in an emergency department setting that was a surrogate, the patient was on the phone with the gay couple that the arrangement was with. Before going into the patient's room, the provider said, ‘I’m too conservative for this shit’ and continued to joke about the situation.” - current student, 4^th^ year, BSN program

Another participant described their perception that patient interactions were viewed more negatively by a member of the healthcare team once they learned that the patient was LGBTQ + .

“After being told a patient’s partner was in the room and they were the same gender, the LNA <licensed nursing assistant> found everything the partner did offensive or wrong. In my experience, the partner was very polite and did nothing offensive.” - current student, 4^th^ year, BSN program

#### Non-affirmation of chosen names and pronouns

This theme describes instances where the participants identify circumstances where they, or other healthcare professionals, used the incorrect name and/or pronouns for a T + patient. The experiences described by participants represented by this theme were described as both accidental and deliberate. Participants tended to describe their non-affirmation behaviors in particular as accidental. One participant described their experience of accidental non-affirmation of a patient’s name or pronouns, differentiating their response and that of the other nurses in clinical settings:

“I have definitely misgendered a patient before by accident but then was corrected and didn’t make that mistake again. I’ve seen nurses not correct themselves and continue to use the wrong name or pronouns, which really bothered me.” - current student, 4^th^ year, BSN program

In occurrences of accidental non-affirmation of chosen names and pronouns, after being corrected by the patient, participants described how they corrected their language and moved forward in the patient interaction:

“I have accidentally misgendered a patient when calling them into a clinical setting. I corrected myself and apologized.” – recent graduate, graduate entry to practice

However, when describing instances of role-modeled healthcare professionals engaging in non-affirmation of patients’ chosen names and pronouns, the perceived intent behind the action was mixed. In some instances, the observed behavior was perceived as accidental; yet in others, the behavior was perceived by participants as purposeful:

“I have witnessed doctors deadnaming patients and using the wrong pronouns even though the patient has expressed their preferred pronouns.” - current student, 3rd year, BSN program

#### Outing patients

This theme describes instances where the participants describe circumstances where they, or other healthcare professionals, outed patients by sharing sexual orientation or gender identity information with individuals who were not part of the care team for that patient. Similar to the theme of *non-affirmation of chosen names and pronouns*, examples under this theme were described as occurring both accidentally and purposefully.

One participant stated that they were unaware that sharing sexual orientation information with people who are not part of the care team was unethical, even when speaking of the experience positively:

“I didn’t realize until I read the question that it was bad to share SOGI < sexual orientation and gender identity> and did it a couple times when I had patients in my maternity rotation. I would get excited and tell my nursing friends about a positive experience with a couple. Looking back I guess it wasn’t necessary to share their sexual orientation.” - recent graduate, graduate entry to practice

Another participant described observing the way that their clinical instructor shared a patient’s gender identity in a manner that was perceived as negative, “A patient was a transgender woman and the nurse told my whole clinical group. The information was not necessary at all and she was very rude about it.” - current student, 2nd year, BSN program.

#### Rejected competency

This theme describes instances where the patients’ competency or the validity of their sexual orientation or gender identity was questioned or rejected. This theme was identified by participants as a perspective held by members of the healthcare team, rather than a view personally held:

“A nurse in a behavioral health unit said that she was ‘appalled’ they ‘allowed’ her (a transgender patient) to go through with transitioning when she’s clearly mentally ill and unstable.”- current student, 4^th^ year, BSN program

A similar view was observed by another participant where a nurse believed a patient was too young to know their gender identity,. “A nursing preceptor from the hospital who seemed to not agree with the fact that the patient identified as the opposite gender when they were 18 years old.” - current student, 3^rd^ year, BSN program

## Discussion

The purpose of this study was to describe the role-modeled behaviors related to the care of LGBTQ + patients in clinical settings, as observed by nursing students and recent graduate nurses. Study participants were asked about the role-modeled healthcare behaviors they observed when caring for LGBTQ + people, reporting that nurses or nursing assistants were observed most frequently engaging in at least one type of stigmatizing behavior. This healthcare professional role was closely followed by providers or other healthcare professionals. A higher frequency of role-modeled stigmatizing behavior by nurses and nursing assistants is expected since nursing students and new graduate nurses are likely to spend the majority of their time in clinical settings working most directly with these roles within the healthcare team. However, members of the healthcare team who, presumably, do not work as closely or as frequently with nursing students and recent graduate nurses were also observed engaging in stigmatizing behaviors by participants. Half of the participants observed LGBTQ + stigmatizing behaviors from multiple healthcare professional roles (*i.e.*, clinical instructors, nurses or nursing assistants, and providers or other healthcare professionals). Late-stage learners among current students and recent nursing graduates reported observing a higher proportion of stigmatizing behaviors across all healthcare professional roles and for most individual behavior types. These findings suggest that as students advance in their education and transition into clinical practice, their exposure to stigmatizing behavior by role models in the clinical setting increases.

A comprehensive review of current literature revealed no studies have examined role-modeled behavior within healthcare settings concerning LGBTQ + stigmatizing behaviors. However, role-modeled behaviors in other healthcare contexts have been studied. Other studies involving undergraduate nursing students found that incidences of implicit and explicit weight bias by healthcare professionals were observed in clinical settings, which they reported as contributing to feelings of internal conflict [34, 35]. Another study found that despite professionalism being taught during their medical education, a third of medical students reported observing unprofessional behavior by physicians in their clinical settings, while 3% observed unprofessional behavior by nurses [42]. Almost 10% of the study’s participants reported incidences of implicit and explicit bias related to race, religion, and other personal characteristics, a lower incidence than observed in the present study of LGBTQ + bias. Another study found that almost 20% of medical students perceived that faculty lacked respect for diversity, although the study did not examine observations made within clinical settings specifically [43]. Further, the study found that racial minority students were more likely to report a perceived lack of respect for diversity by faculty, which led the study’s authors to hypothesize that being a member of the marginalized group might increase awareness or recall of bias-related incidents. Given that approximately 11% of our sample identified as LGBTQ + , a corresponding increase in awareness or memory of the bias-related incidents is possible.

Comparable literature on role modeling in healthcare professions, particularly nursing, is scarce as research predominantly focuses on clinical skills and orientation to professional roles, with a focus solely on same-profession faculty and assigned mentors [32, 44]. This study, describing how the behavior of healthcare professionals across the healthcare team is observed by nursing students and recent graduate nurses, points to an opportunity to expand existing notions of who acts as role models for nursing students and recent graduate nurses, and to examine how multidisciplinary roles contribute to either the normalization of stigmatizing or inclusive care practices toward LGBTQ + patients.

When participants described their stigmatizing behaviors towards LGBTQ + patients, almost a third reported that they had engaged in one or more behaviors. This result was noteworthy because almost 88% of participants reported having an LGBTQ + close friend or family member, a considerably higher percentage than reported in a recent study of the US general population, where only 51% of adults knew someone who is LGBTQ + [45]. Conversely, just over a tenth of our sample self-identified as LGBTQ + , although this is lower than recent survey findings of the US population where 20% of adults aged 18–23 years, similar to the age range of the present sample, identified as LGBTQ + [46]. The present sample also showed broadly positive attitudes toward LGBTQ + people. However, when analyzing the subscales, participants’ attitudes were more negative toward T + people. This may explain why the most frequent type of LGBTQ + stigmatizing behavior that participants reported engaging in was misgendering a patient. Similarly, in the examples of LGBTQ + stigma provided by open-text responses gender identity and T + people were consistently mentioned. While causality cannot be determined, role-modeled LGBTQ + stigma may be a contributing factor as the most common type of stigmatizing behavior role modeled by healthcare professionals was also related to gender identity and T + people.

It is also of note that all of the themes included examples of stigma that occurred in the context of conversations among healthcare professionals, outside of direct patient interaction. These role-modeled stigmatizing behaviors from multiple parts of the healthcare team reinforce these behaviors as acceptable, normalizing prejudice as part of the healthcare culture. The observations of these behaviors among various healthcare professionals outside of direct patient interactions place pressure on students and recent graduates to participate, as the sense of belonging is important for students [47] and power differentials impair students’ ability to speak out [48, 49].

### Limitations

It is important to note that the sample composition introduces several potential sources of bias. The present study was limited to only 73 participants, who were fairly homogenous concerning gender, sexuality, race, and ethnicity. With over 255,000 students enrolled in undergraduate nursing programs in 2023, this study’s sample size is very small [36]. In particular, the subgroup of recent graduates was comprised of only eight participants. Therefore, the findings are not generalizable to the general population of nursing students and recent graduates, and should be viewed as exploratory. The findings also are limited in their generalizability to other countries where there may be differences in population characteristics, nursing educational requirements, and societal attitudes toward LGBTQ + people [50, 51]. Additionally, the measurement of observed LGBTQ + stigmatizing behaviors was developed for this study, as no other measurement scales or instruments have been developed. The items have not been evaluated for validity or reliability. The lack of psychometric evaluation may result in measurement error and should be explored in future studies.

Social desirability bias may have played a part in the quality of responses from participants. Evidence of this can be observed in how examples of participants’ LGBTQ + stigmatizing behaviors were framed when compared to LGBTQ + stigmatizing behaviors observed among other healthcare professionals. Students described their stigmatizing behaviors as accidental and often stated that they took responsibility immediately. Examples of LGBTQ + stigmatizing behaviors observed among other healthcare professionals were more often framed as intentional or malicious. In addition to the desire for some participants to provide socially acceptable answers related to their behaviors, they may also feel personally connected to some healthcare professionals, which could result in lower reporting and a more forgiving attitude toward those individuals [52]. Further, the setting where these role-modeled behaviors were observed was not measured and cannot be inferred. This limits the ability to examine potential differences, such as inpatient versus outpatient environments, or intensive care versus medical surgical floors.

### Future directions

Overall, few studies can serve as a comparable context for our findings, as existing studies either present findings related to LGBTQ + people as a single group, or solely focus on T + people [26, 53, 54]. Fewer still have studied LGBTQ + stigmatizing behaviors observed by clinicians/students in healthcare settings, instead reporting their LGBTQ + knowledge and attitudes. One qualitative study interviewed healthcare professionals about LGBTQ + stigma observed in clinical settings and found more behaviors related to gender identity and T + individual than LGB individuals [55]. Another study that interviewed LGBTQ + patients and healthcare providers in multiple countries described that some healthcare providers felt as though the care of LGB people was adequate or no longer a concern, but that T + care was the “latest big thing” [56]. While understanding the knowledge level of healthcare professionals and their attitudes is an important context in efforts to improve LGBTQ + healthcare experiences, the continued use of measures and study designs that conflate LGBTQ + people and their health needs into a monolithic group obscures important deficits in the preparedness of nurses and other healthcare professionals. The lack of nuanced measurement inhibits the development and rigorous evaluation of interventions aimed at improving the healthcare experiences of T + people, in particular. It also can leave healthcare professionals under a possibly false assumption that their care of LGBTQ + people is adequate since the gaps between constructs as they relate to LGB and T + people separately are obscured.

Further research needs to be conducted on the effects of role-modeling and peer pressure on nurses’ abilities to provide care to minorities and vulnerable populations. This includes research on prelicensure nursing students' and new graduate nurses’ susceptibility to peer pressure. Understanding workplace culture, including the distinct forms of social pressure that arise from peer dynamics and power differentials, can inform better interventions to mitigate these influences. Such efforts are essential to prevent individuals in perceived positions of power from unduly influencing students and new graduates, who may be in positions of relative vulnerability. However, it is important to examine these phenomena based on the distinct experiences of each group: current students within clinical rotations, and new graduates entering the workforce. This will contribute to perpetuating a positive learning culture for nursing students and new graduate nurses.

## Conclusion

Many nursing students and recent graduates observe role models, from various disciplines within the healthcare team, engage in stigmatizing behaviors toward LGBTQ + patients, most notably T + people. Despite positive LGBTQ + attitudes and having close friends and family who are part of the LGBTQ + community, students themselves also engage in stigmatizing behaviors in clinical settings. Continued efforts to address implicit and explicit LGBTQ + bias in healthcare settings should be applied in both didactic and clinical settings to address role-modeled behaviors and subsequent socialization of students and recent graduates. The incorporation of role modeling into research to reduce LGBTQ + stigma in healthcare provides an important perspective that can improve healthcare professional education. Advancement of this area of research can lead to more supportive healthcare institutions where LGBTQ + people can focus on healing in safe, inclusive spaces. Future research should also ensure that outcomes can be measured with both LGB and T + populations separately as the present study identified that participants had worse attitudes toward T + people than LGB and the majority of stigmatizing behaviors noted were related to gender identity and T + individuals. 

## Data Availability

The datasets generated and/or analysed during the current study are not publicly available due conditions of the data management agreement to protect participant privacy. The data that support the findings of this study are available upon reasonable request from the authors.

## Abbreviation

LGBTQ +: Lesbian, gay, bisexual, transgender, queer, and other sexually and gender-diverse people
